# A Standardized Project Gutenberg Corpus for Statistical Analysis of Natural Language and Quantitative Linguistics

**DOI:** 10.3390/e22010126

**Published:** 2020-01-20

**Authors:** Martin Gerlach, Francesc Font-Clos

**Affiliations:** 1 Department of Chemical and Biological Engineering, Northwestern University, Evanston, IL 60208, USA; martin.gerlach@northwestern.edu; 2 Center for Complexity and Biosystems, Department of Physics, University of Milan, 20133 Milano, Italy

**Keywords:** Project Gutenberg, Jensen–Shannon divergence, reproducibility, quantitative linguistics, natural language processing

## Abstract

The use of Project Gutenberg (PG) as a text corpus has been extremely popular in statistical analysis of language for more than 25 years. However, in contrast to other major linguistic datasets of similar importance, no consensual full version of PG exists to date. In fact, most PG studies so far either consider only a small number of manually selected books, leading to potential biased subsets, or employ vastly different pre-processing strategies (often specified in insufficient details), raising concerns regarding the reproducibility of published results. In order to address these shortcomings, here we present the Standardized Project Gutenberg Corpus (SPGC), an open science approach to a curated version of the complete PG data containing more than 50,000 books and more than $3\times 10^{9}$ word-tokens. Using different sources of annotated metadata, we not only provide a broad characterization of the content of PG, but also show different examples highlighting the potential of SPGC for investigating language variability across time, subjects, and authors. We publish our methodology in detail, the code to download and process the data, as well as the obtained corpus itself on three different levels of granularity (raw text, timeseries of word tokens, and counts of words). In this way, we provide a reproducible, pre-processed, full-size version of Project Gutenberg as a new scientific resource for corpus linguistics, natural language processing, and information retrieval.

## 1. Introduction

Analysis of natural language from a complex systems perspective has provided new insights into statistical properties of language, such as statistical laws [1,2,3,4,5,6,7,8,9], networks [10,11,12,13,14], language change [15,16,17,18,19,20], quantification of information content [21,22,23,24], or the role of syntactic structures [25] or punctuation [26], etc. In particular, the availability of new and large publicly available datasets such as the google-ngram data [27], the full Wikipedia dataset [28,29], or Twitter [30] opened the door for new large-scale quantitative approaches. One of the main drawbacks of these datasets, however, is the lack of “purity” of the samples resulting from the fact that (i) the composition of the dataset is largely unknown (google-ngram data, see [31,32]); (ii) the texts are a mixture of different authors (Wikipedia); or (iii) the texts are extremely short (Twitter). One approach to ensure large homogeneous samples of data is to analyze literary books – the most popular being from Project Gutenberg (PG) [33] due to their free availability.

Data from PG has been used in numerous cases to quantify statistical properties of natural language. In fact, the statistical analysis of texts in the framework of complex systems or quantitative linguistics is not conceivable without the books from PG. Already in the 1990s the seminal works by Ebeling et al. [34] and Schurman and Grassberger [35] used up to 100 books from PG in the study of long-range correlations and Baayen [36] investigated the growth curve of the vocabulary. Subsequently, PG has become an indispensable resource for the quantitative analysis of language investigating, e.g., universal properties (such as correlations [37] or scale-free nature of the word-frequency distribution [38,39,40]) or aspects related to genres [41] or emotions [42]. While we acknowledge that PG has so far been of great use to the community, we also find that it has been handled in a careless and unsystematic way. Our criticisms can be summarized in two points. First, the majority of studies only consider a small subset (typically not more than 20 books) from the more than 50,000 books available in PG. More importantly, the subsets often contain the same manually selected books such as the work “Moby Dick” which can be found in virtually any study using PG data. Thus different works potentially employ biased and correlated subsets. While in some occasions a small set of well-selected books can be sufficient to answer particular linguistic questions, we believe that the enduring task of manually downloading and processing individual PG books has been the major bound to the number of books used in many PG studies. In the study of linguistic laws and its associated exponents, for instance, the variability they display [38,43] can be hard to grasp with reduced samples of PG. Even more clear is the case of double-power laws in Zipfs’ law [44], which is only observed in very large samples and has been conjectured to be an artefact of text mixing [39]. Second, different studies use different filtering techniques to mine, parse, select, tokenize, and clean the data or do not describe the methodological steps in sufficient detail. As a result, two studies using the supposedly same PG data might end up with somewhat different datasets. Taken together, these limitations raise concerns about the replicability and generalizability of previous and future studies. In order to ensure the latter, it is pertinent to make corpora widely available in a standardized format. While this has been done for many textual datasets in machine learning (e.g., the UCI machine learning repository [45]) and diachronic corpora for studying language change (e.g., The Corpus of Contemporary American English [46]), such efforts have so far been absent for data from PG.

Here, we address these issues by presenting a standardized version of the complete Project Gutenberg data—the Standardized Project Gutenberg Corpus (SPGC)—containing more than 50,000 books and more than $3\times 10^{9}$ word-tokens. We provide a framework to automatically download, filter, and process the raw data on three different levels of granularity: (i) the raw text; (ii) a filtered timeseries of word-tokens, and (iii) a list of occurrences of words. We further annotate each book with metadata about language, author (name and year of birth/death), and genre as provided by Project Gutenberg records as well as through collaborative tagging (so-called bookshelves), and show that the latter has more desirable properties such as low overlap between categories. We exemplify the potential of the SPGC by studying its variability in terms of Jensen–Shannon divergence across authors, time and genres. In contrast to standard corpora such as the British National Corpus [47] or the Corpus of Contemporary American English [46], the new Standardized Project Gutenberg Corpus is decentralized, dynamic and multi-lingual. The SPGC is decentralized in the sense that anyone can recreate it from scratch in their computer executing a simple python script. The SPGC is dynamic in the sense that, as new books are added to PG, the SPGC incorporates them immediately, and users can update their local copies with ease. This removes the classic centralized dependency problem, where a resource is initially generated by an individual or institution and initially maintained for certain period of time, after which the resource is no longer updated and remains “frozen” in the past. Finally, the SPGC is multi-lingual because it is not restricted to any language, it simply incorporates all content available in PG (see Section 2.3 for details). These characteristics are in contrast with some standard corpus linguistics practices [48,49], where a corpus is seen as a fixed collection of texts, stored in a way that allows for certain queries to be made, usually in an interactive way. While these corpora are well-suited –logically– for computational linguists interested in specific linguistic patterns, they are less useful for researchers of other disciplines such as physicists interested in long-range correlations in texts or computer scientists willing to train their language models on large corpora. In order to be compatible with a standard corpus model, and to ensure reproducibility of our results, we also provide a static time-stamped version of the corpus, SPGC-2018-07-18 (https://doi.org/10.5281/zenodo.2422560). In summary, we hope that the SPGC will lead to an increase in the availability and reliability of the PG data in the statistical and quantitative analysis of language.

## 2. Results

### 2.1. Data Acquisition

Project Gutenberg is a digital library founded in 1971 which archives cultural works uploaded by volunteers. The collection primarily consists of copyright-free literary works (books), currently more than 50,000, and is intended as a resource for readers to enjoy literary works that have entered the public domain. Thus the simplest way for a user to interact with PG is through its website, which provides a search interface, category listings, etc. to facilitate locating particular books of interest. Users can then read them online for free, or download them as plain text or ebook format. While such a manual strategy might suffice to download tens or hundreds of books (given the patience), it does not reasonably scale to the complete size of the PG data with more than 50,000 books.

Our approach consists of downloading the full PG data automatically through a local mirror, see Project Gutenberg’s Information About Robot Access page (https://www.gutenberg.org/wiki/Gutenberg:Information_About_Robot_Access_to_our_Pages) for details. We keep most technical details “under the hood” and instead present a simple, well structured solution to acquire all of PG with a single command. In addition to the book’s data, our pipeline automatically retrieves two different datasets containing annotations about PG books. The first set of metadata is provided by the person who uploads the book, and contains information about the author (name, year of birth, year of death), language of the text, subject categories, and number of downloads. The second set of metadata, the so-called bookshelves, provide a categorization of books into collections such as “Art” or “Fantasy”, in analogy to the process of collaborative tagging [50].

### 2.2. Data Processing

**Figure 1:**
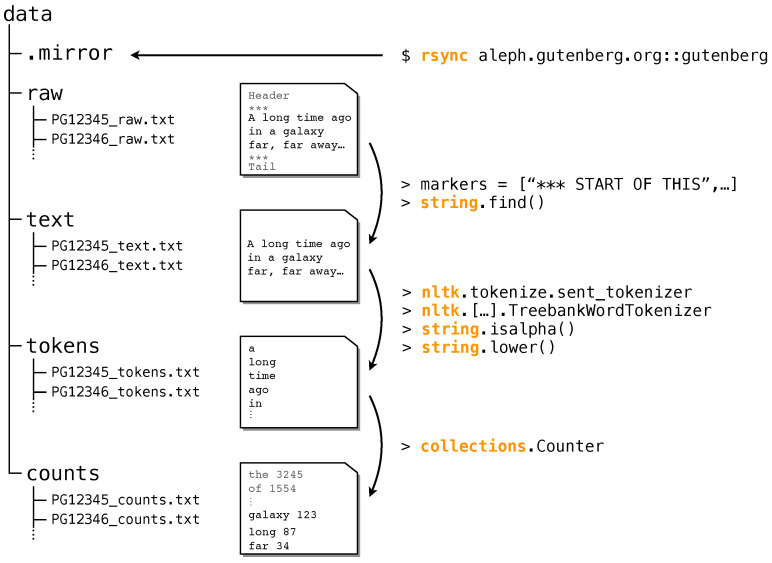
Sketch of the pre-processing pipeline of the Project Gutenberg (PG) data. The folder structure (**left**) organizes each PG book on four different levels of granularity, see example books (**middle**): raw, text, tokens, and counts. On the right we show the basic python commands used in the pre-processing.

In this section, we briefly describe all steps we took to obtain the corpus from the raw data (Figure 1), for details see Section 4. The processing (as of 18 July 2018) yields data for 55,905 books on four different levels of granularity:

- *Raw* data: We download all books and save them according to their PG-ID. We eliminate duplicated entries and entries not in UTF-8 encoding.
- *Text* data: We remove all headers and boiler plate text, see Methods for details.
- *Token* data: We tokenize the text data using the tokenizer from NLTK [51]. This yields a time series of tokens without punctuation, etc.
- *Count* data: We count the number of occurrences of each word-type. This yields a list of tuples (*w*,$n_{w}$), where *w* is word-type *w* and $n_{w}$ is the number of occurrences.

### 2.3. Data Description

**Figure 2:**
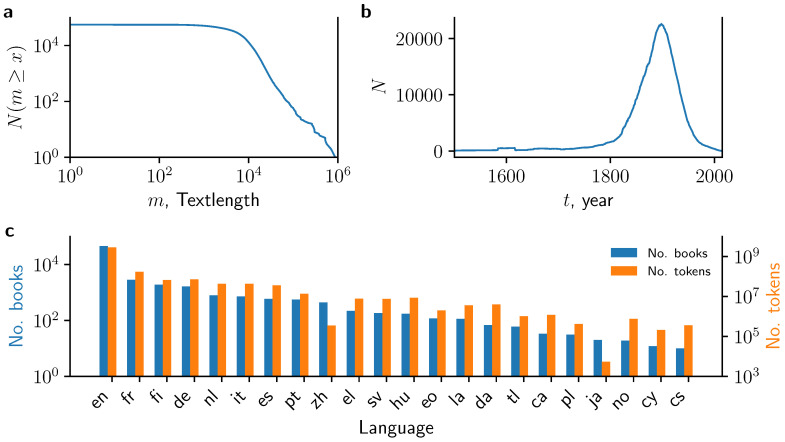
Basic summary statistics from the processed PG data. (**a**) Number of books with a text length larger than *m*; (**b**) Number of books which are compatible with being published in year *t*, i.e., year of author’s birth is 20 years prior and year of author’s death is after *t*; (**c**) Number of books (left axis) and number of tokens (right axis) which are assigned to a given language based on the metadata. en: English, fr: French, fi: Finnish, de: German, nl: Dutch, it: Italian, es: Spanish, pt: Portuguese, zh: Chinese, el: Greek, sv: Swedish, hu: Hungarian, eo: Esperanto, la: Latin, da: Danish, tl: Tagalog, ca: Catalan, pl: Polish, ja: Japanese, no: Norwegian, cy: Welsh, cs: Czech.

We provide a broad characterization of the PG books in terms of their length, language and (when available) inferred date of publication in Figure 2. One of the main reason for the popularity of books from PG is their long text length, which yields large coherent statistical samples without potentially introducing confounding factors originating from, e.g., the mixing of different texts [39]. The length of most PG books exceeds $m=10^{4}$ word tokens (Figure 2a) which is larger than typical documents from most web resources. In fact, the distribution shows a heavy-tail for large values of *m*. Thus we find a substantial fraction of books having more than $10^{5}$ word tokens. Many recent applications in quantitative linguistics aim at tracing diachronic changes. While the metadata does not provide the year of the first publication of each book, we approximate the number of PG books published in year *t* as the number of PG books for which the author’s year of birth is $t_{\mathrm{birth}}+20<t$ and the author’s year of death is $t<t_{\mathrm{death}}$ (Figure 2b). This reveals that the vast majority of books were first published around the year 1900, however, with a substantial number of books between 1800 and 2000. Part of this is known to be a consequence of the Copyright Term Extension Act of 1998 which, sadly, has prevented books published after 1923 to enter the public domain so far. If no further copyright extensions laws are passed in the future, then this situation will be gradually alleviated year after year, as books published in 1923 will enter the public domain on 1 January 2019, and so on.

While most contemporary textual datasets are in English, the SPGC provides a rich resource to study other languages. Using metadata provided by PG, we find that 81% of the books are tagged as written in English, followed by French (5%, 2864 books), Finnish (3.3%, 1903 books) and German (2.8%, 1644 books). In total, we find books written in 56 different languages, with three (13) languages besides English with more than 1000 (100) books each (Figure 2c). The size of the English corpus is $2.8\times 10^{9}$ tokens, which is more than one order of magnitude larger than the British National Corpus ($10^{8}$ tokens). The second-largest language corpus is made up of French books with $>10^{8}$ tokens. Notably, there are six other languages (Finnish, German, Dutch, Italian, Spanish, and Portuguese) that contain $>10^{7}$ tokens and still another eight languages (Greek, Swedish, Hungarian, Esperanto, Latin, Danish, Tagalog, and Catalan) that contain $>10^{6}$ tokens.

**Figure 3:**
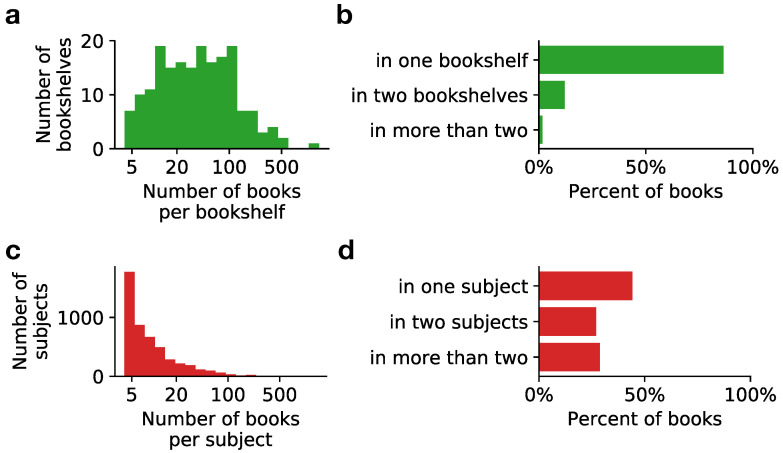
Comparison between bookshelf labels (top, green) and subject labels (bottom, red). (**a**,**c**) Number of labels with a given number of books; (**b**,**d**) Fraction of books with a given number of labels.

In addition to the “hard-facts” metadata (such as language, time of publication), the SPGC also contains manually annotated topical labels for individual books. These labels allow not only the study of topical variability, but they are also of practical importance for assessing the quality of machine learning applications in Information Retrieval, such as text classification or topic modeling [52]. We consider two sets of topical labels: labels obtained from PG’s metadata “subject” field, which we call *subject labels*; and labels obtained by parsing PG’s website bookshelf pages, which we call *bookshelf labels*. Table 1 shows that there is certain overlap in the most common labels between the two sets (e.g., Science Fiction or Historical Fiction), but a more detailed analysis of how labels are assigned to books reveals substantial differences (Figure 3). First, subject labels display a very uneven distribution of the number of books per label. That is, most of the subject labels are assigned to very few books (less than 10), with only few subject labels assigned to many books. In comparison, bookshelf labels are more evenly distributed: most of them are assigned to between 10 and 100 books (Figure 3a,c). More importantly, the overlap in the assignment of labels to individual books is much smaller for the bookshelf labels (Figure 3b,d): While roughly 50% of the PG books are tagged with two or more subject labels, up to 85% of books are tagged with a unique bookshelf label. This indicates that the bookshelf labels are more informative because they constitute broader categories and provide a unique assignment of labels to books, and are thus better suited for practical applications such as text classification.

### 2.4. Quantifying Variability in the Corpus

In order to highlight the potential of the SPGC for quantitative analysis of language, we quantify the degree of variability in the statistics of word frequencies across labels, authors, and time. For this, we measure the distance between books *i* and *j* using the well-known Jensen–Shannon divergence [53], $D_{i,j}$, with $D_{i,j}=0$ if the two books are exactly equal in terms of frequencies, and $D_{i,j}=1$ if they are maximally different, i.e., they do not have a single word in common, see Methods for details.

#### 2.4.1. Labels

**Figure 4:**
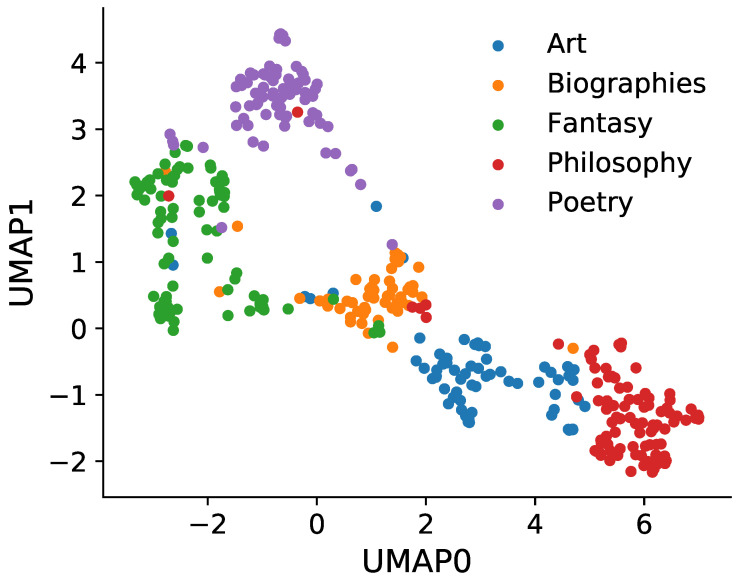
2-dimensional embedding shows clustering of books from the same bookshelf. Approximate visualization of the pair-wise distances between 370 PG books using Uniform Manifold Approximation and Projection (UMAP) (for details, see Section 4). Each dot corresponds to one book colored according to the bookshelf membership.

We select the 370 books tagged with one of the following bookshelf labels: Art, Biographies, Fantasy, Philosophy and Poetry. After calculating distances $D_{i,j}$ between all pairs of books, in Figure 4 we show an approximate 2-dimensional embedding (Uniform Manifold Approximation and Projection (UMAP), see [54] and Methods for details) in order to visualize which books are more similar to each other. Indeed, we find that books from the same bookshelf tend to cluster together and are well-separated from books belonging to other bookshelves. This example demonstrates the usefulness of the bookshelf labels and that they reflect the topical variability encoded in the statistics of word frequencies.

#### 2.4.2. Authors

**Figure 5:**
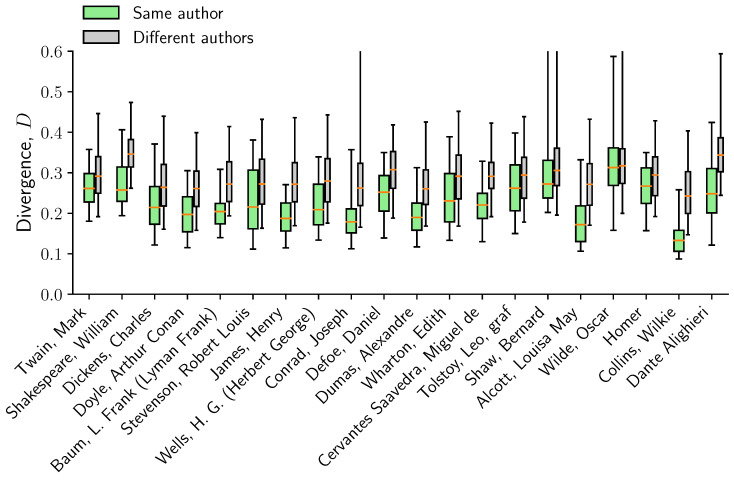
Distance between books from the same author is significantly smaller than distance between books from different authors. For each author, the boxplots shows the 5,25,50,75,95-percentile of the distribution of distances from 1000 pairs of books from the same author (**green**) and to a different author (**gray**).

We select all books from the 20 most prolific authors (selected from the authors of the 100 most downloaded books in order to avoid authors such as “Anonymous”). For each author, we draw 1000 pairs of books (i,j) from the same author and compare the distance $D_{i,j}$ with 1000 pairs $\left(i,j^{\prime }\right)$ where $j^{\prime }$ comes from a different author. We observe that the distance between books from the same author is consistently smaller than for two books from different authors – not only in terms of the median, but also in terms of a much smaller spread in the values of $D_{i,j}$ (Figure 5). This consistent variability across authors suggest the potential applicability in the study of stylistic differences, such as in problems of authorship attribution [55,56].

#### 2.4.3. Time

**Figure 6:**
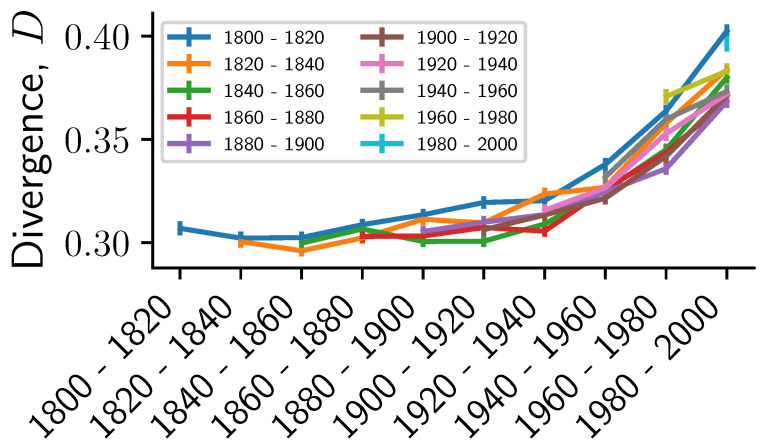
Distance between books increases with their time separation. Average and standard error of the distance between 1000 pairs of books, where the two books in each pair is drawn from two different 20-year time intervals. We fix the first interval and continuously increase the second time interval.

We compare the distance $D_{i,j}$ between pairs of books i,j taken each from a 20-year time period $t_{i},t_{j}\in \left\{1800-1820,\ldots ,1980-2000\right\}$. In Figure 6, we show the distance between two time windows $D_{t_{i},t_{j}}$ by averaging over each 1000 pairs of books. We observe that the average distance increases with increasing separation between the time periods. However, we emphasize that we only observe a substantial increase in $D_{t_{i},t_{j}}$ for large separation between $t_{i}$ and $t_{j}$ and later time periods (after 1900). This could be caused by the rough approximation of the publication year and a potential change in composition of the SPGC after 1930 due to copyright laws. In fact, the observed effects are likely a mixture of temporal and topical variability, because the topical composition of PG over time is certainly not uniform. This suggests the limited applicability of PG books for diachronic studies without further filtering (such as subject/bookshelf). Other resources such as the COHA corpus might be more adequate in this case, although potentially a more genre-balanced version of SPGC could be created using the provided metadata.

## 3. Discussion

We have presented the Standardized Project Gutenberg Corpus (SPGC), a decentralized, dynamic multilingual corpus containing more than 50,000 books from more than 20 languages. Combining the textual data with metadata from two different sources we provided not only a characterization of the content of the full PG data but also showed three examples for resolving language variability across subject categories, authors, and time. As part of this work, we provide the code for all pre-processing steps necessary to obtain a full local copy of the PG data. We also provide a static or ‘frozen’ version of the corpus, SPGC-2018-07-18, which ensures reproducibility of our results and can be downloaded at https://doi.org/10.5281/zenodo.2422560.

We believe that the SPGC will be a first step towards a more rigorous approach for using Project Gutenberg as a scientific resource. A detailed account of each step in the pre-processing, accompanied by the corresponding code, are necessary requirements that will help ensure replicability in the statistical analysis of language and quantitative linguistics, especially in view of the crisis in reproducibility and replicability reported in other fields [57,58,59]. From a practical point of view, the availability of this resource in terms of the code and the frozen dataset will certainly allow for an easier access to PG data, in turn facilitating the usage of larger and less biased datasets increasing the statistical power of future analysis.

We want to highlight the challenges of the SPGC in particular and PG in general, some of which can hopefully be addressed in the future. First, the PG data only contains copyright-free books. As a result the number of books published after 1930s is comparably small. However, in the future this can be expected to change as copyright for many books will run out and the PG data is continuously growing. This highlights the importance of using a dynamic corpus model that will by default incorporate all new books when the corpus is generated for the first time. Second, the annotation about the books is incomplete, and some books might be duplicated. For example, the metadata lacks the exact date when a book was published, hindering the usage of the PG data for diachronic studies. Different editions of the same book might have been given a different PG identifier, and so they are all included in PG and thus in SPGC. Third, the composition of SPGC is heterogeneous, mixing different genres. However, the availability of document labels from the bookshelf metadata allows for systematic control of corpus composition. For example, it is easy to restrict to or exclude individual genres such as “Poetry”.

From a practical perspective, the SPGC has a strong potential to become a complementary resource in applications ranging from computational linguistics to machine learning. A clear limitation of SPGC is that it was designed to fit a wide range use cases, and so the pre-processing and data-selection choices are sub-optimal in many specific cases. However, the modular design of the code allows for researches to modify such choices with ease, and data can always be filtered a posteriori, but not the other way around. Choices are unavoidable, but it is only by providing the full code and data that these choices can later be tailored to specific needs. Overall, we believe the benefits of a standardized version of PG out-weight its potential limitations.

We emphasize that the SPGC contains thousands of annotated books in multiple languages even beyond the Indo-European language family. There is an increasing interest in quantitative linguistics in studies beyond the English language. In the framework of culturomics, texts could be annotated and weighted by additional metadata, e.g., in terms of their ‘success’ measure as the number of readers [60] or number of PG downloads. For example, it could be expected that the impact of Carroll’s “Alice in Wonderland” is larger than that of the “CIA Factbook 1990”. Furthermore, with an increase in the quality of the metadata, the identification of the same book in different languages might allow for the construction of high-quality parallel corpora used in, e.g., translation tasks. Finally, in applications of Information Retrieval, metadata labels can be used to evaluate machine learning algorithms for classification and prediction. These and other applications might require additional pre-processing steps (such as stemming) but which could make use of SPGC as a starting point.

In summary, we believe that the SPGC is a first step towards a better usage of PG in scientific studies, and hope that its decentralized, dynamic and multi-lingual nature will lead to further collaborative interdisciplinary approaches to quantitative linguistics.

## 4. Materials and Methods

### 4.1. Running the Code

The simplest way to get a local copy of the PG database, with standardized, homogeneous pre-processing, is to clone the git repository

$ git clone git@github.com:pgcorpus/gutenberg.git

and enter the newly created directory. To get the data, simply run:

$ python get_data.py

This will download all available PG books in a hidden ‘.mirror’ folder and symlink in the more convenient ‘data/raw’ folder. To actually process the data, that is, to remove boiler-plate text, tokenize texts, filter and lowercase tokens, and count word type occurrence, it suffices to run

This will download all available PG books in a hidden ‘.mirror’ folder and symlink in the more convenient ‘data/raw’ folder. To actually process the data, that is, to remove boiler-plate text, tokenize texts, filter and lowercase tokens, and count word type occurrence, it suffices to run

$ python process_data.py

which will fill in the rest of directories inside ‘data’. We use ‘rsync’ to keep an updated local mirror of aleph.gutenberg.org::gutenberg. Some PG book identifiers are stored in more than one location in PG’s server. In these cases, we only keep the latest, most up-to-date version. We do not remove duplicated entries on the basis of book metadata or content. To eliminate boiler-plate text that does not pertain to the books themselves, we use a list of known markers (code adapted from https://github.com/c-w/gutenberg/blob/master/gutenberg/cleanup/strip_headers.py).

### 4.2. Preprocessing

Texts are tokenized via NLPToolkit [51]. In particular, we set the ‘TreebankWordTokenizer‘ as the default choice, but this can be changed at will. Tokenization works better when the language of the text being analyzed is known. Since the metadata contains a language field for every downloaded book, we pass this information onto the tokenizer. If the language field contains multiple languages (≈0.3% of the books), we use the first entry. We only keep tokens composed entirely of alphabetic characters (including accented characters), removing those that contain digits or other symbols. Notice that this is done after tokenization, which correctly handles apostrophes, hyphens, etc. This constitutes a conservative approach to avoid artifacts, for example originating from the digitization process. While one might want to also include tokens with numeric characters in order to keep mentions of, e.g., years, the downside of this approach would be a substantial number of occurrences of undesirable page and chapter numbers. However, we note that the modularized filtering can be easily customized (and extended) to incorporate also other aspects such as stemming as there is no one-size-fits all solution to each individual application. Furthermore, all tokens are lower-cased. While this has undesired consequences in some cases (e.g., some proper nouns can be confounded with unrelated common nouns after being lower-cased), it is a simple and effective way of handling words capitalized after full stop or in dialogues, which would otherwise be (incorrectly) considered different words from their lowercase standard form. We acknowledge that our preprocessing choices might not fit well specific use cases, but they have been designed to favour precision over recall. For instance, we find it preferable to miss mentions of years while ensuring that no page or chapter numbers are included in the final dataset. Notice that the alternative, that of building and additional piece of software that correctly distinguishes these two cases, is in itself complicated, has to handle several edge cases, and ultimately incurs in additional choices.

### 4.3. Jensen–Shannon Divergence

We use Jensen–Shannon divergence (JSD, [53,61,62]) as a divergence measure between PG books. JSD is an information-theory based divergence measure which, given two symbolic sequences with frequency distributions encoded in vectors p,q can be simply defined as

(1) $$D\left(p,q\right)=H\left(\frac{p+q}{2}\right)-\frac{1}{2}H\left(p\right)-\frac{1}{2}H\left(q\right)$$

where H(p) denotes the standard Shannon entropy, $H\left(p\right)=-\sum_{i}p_{i}\log p_{i}$. In simple and general terms, JSD quantifies how similar two symbolic sequences are on the basis of how frequent or infrequent each symbol is in the two sequences. In our case, this translates to measuring the similarity between two books via the frequencies of their word types. The logarithmic term in the expression of the entropy H(p), however, ensures that the measure is not dominated by high-frequency words, as would happen otherwise, but instead is dependent on differences in frequency along the whole spectrum of usage, from very common to very uncommon words. Therefore, JSD is specially suitable for symbolic sequences that display long tails in the distribution of frequencies, as is the case in natural language. Notice that the distance between one book and a randomly shuffled version of it is exactly 0, from the JSD point of view. This drawback can be alleviated by using JSD on *n*-grams counts of higher order, that is, taking into account the frequency of pairs of words or bigrams, and so on. However, we do not take this route here since it has the undesirable consequence of exponentially increasing the number of features. For a more technical discussion about JSD and related measures, see [53].

### 4.4. 2-Dimensional Embedding

We use *Uniform Manifold Approximation and Projection* (UMAP, [54]) for visualization purposes in Figure 4. UMAP is a manifold-learning technique with strong mathematical foundations that aims to preserve both local and global topological structures, see [54] for details. In simple terms, UMAP and other manifold-learning algorithms aim at finding a good low-dimensional representation of a high-dimensional dataset. To do so, UMAP finds the best manifold that preserves topological structures of the data at different scales, again see [54] for details. We used normalized counts data as the input data, with word types playing the role of dimensions (features) and books playing the role of points (samples). Distance between points was computed using the Jensen–Shannon divergence [53]. The end result is the 2-dimensional projection shown in Figure 4. Notice that subject labels were not passed to UMAP, so the observed clustering demonstrates that the statistics of word frequencies encode and reflect the manually-assigned labels.

### 4.5. Data Availability

The code that we make available as part of this work allows to download and process all available Project Gutenberg books, facilitating the task of keeping an up-to-date and homogeneously processed dataset of a continuously growing resource. In fact, new books are added to Project Gutenberg daily. An unwanted consequence of this feature, however, is that two local versions of the SPGC might differ if they were last updated on different dates. To facilitate and promote reproducibility of our results and possible subsequent analysis, we provide a ’frozen’ copy of the SPGC, last updated on 18 July, 2018, containing 55,905 PG books. All statistics and figures reported on this manuscript are based on this version of the data. This data is available at https://doi.org/10.5281/zenodo.2422560.

### 4.6. Computational Requirements

The ’frozen’ dataset of all 55,905 books and all levels of granularity has a size of 65 GB. However, focusing only on the one-gram counts requires only 3.6 GB. Running the pre-processing pipeline of the ’frozen’ data took 8 h (without parallelization) on an CPU running at 3.40 GHz.

### 4.7. Code Availability

Python 3.6 code to download and pre-process all PG books can be obtained at https://github.com/pgcorpus/gutenberg, while python-based jupyter notebooks that reproduce the results of this manuscript can be obtained at https://github.com/pgcorpus/gutenberg-analysis.

## Figures and Tables

**Table 1:** Examples for the names of labels and the number of assigned books from bookshelves (left) and subjects (right) metadata.

Rank	Books	Bookshelf	Rank	Books	Subject
1	1341	Science Fiction	1	2006	Fiction
2	509	Children’s Book Series	2	1823	Short stories
3	493	Punch	3	1647	Science fiction
4	426	Bestsellers, American, 1895-1923	3	1647	Science fiction
5	383	Historical Fiction	5	746	Historical fiction
6	374	World War I	6	708	Love stories
7	339	Children’s Fiction	7	690	Poetry
…	…	…	…	…	…
47	94	Slavery	47	190	Short stories, American
48	92	Western	48	188	Science – Periodicals
49	90	Judaism	49	183	American poetry
50	86	Scientific American	50	180	Drama
51	84	Pirates, Buccaneers, Corsairs, etc.	51	165	Paris (France) – Fiction
52	83	Astounding Stories	52	163	Fantasy literature
53	83	Harper’s Young People	53	162	Orphans – Fiction
…	…	…	…	…	…
97	37	Animals-Wild-Reptiles and Amphibians	97	100	Scotland – Periodicals
98	37	Short Stories	98	98	Horror tales
99	36	Continental Monthly	99	97	Canada – Fiction
100	35	Architecture	100	97	France – Court and courtiers
101	35	Bahá’í Faith	101	96	Social classes – Fiction
102	34	Precursors of Science Fiction	102	95	Courtship – Fiction
103	33	Physics	103	95	Seafaring life – Juvenile fiction
…	…	…	…	…	…
